# Supplementary material for: Division of labor and collective functionality in Escherichia coli under acid stress
Source: Commun Biol. 2022 Apr 7;5:327. doi: 10.1038/s42003-022-03281-4 (PMC8989999; doi:10.1038/s42003-022-03281-4)
Supplement: Supplementary file 2 — Supplementary Information [file 42003_2022_3281_MOESM2_ESM.pdf]

Supplementary information

Division of labor and collective functionality in *Escherichia coli* under acid stress

Sophie Brameyer, Kilian Schumacher, Sonja Kuppermann, and Kirsten Jung*

Department of Biology, Microbiology, Ludwig-Maximilians-Universität München, Martinsried, Germany

*Corresponding author:
Prof. Dr. Kirsten Jung
Ludwig-Maximilians-Universität München
Microbiology
Großhaderner Str. 2-4
82152 Martinsried
Phone: + 49 89/2180-74500
Fax: + 49 89/2180-74520
E-Mail: jung@lmu.de

Supplementary Table 1: Quantification of fluorescent microscopic images of different *E. coli* strains during acidic conditions. Quantified noise and mean of relative fluorescence intensity (RF) were calculated for 1,000 cells per condition and time point. Cells were cultivated as described in Fig. 2a. Single-cell fluorescence intensity was acquired by microscopy and the use of the MicrobeJ plugin of the ImageJ software. Relative fluorescence intensity (RF) is the first value. Noise, standard deviation/mean of log-transformed values in the second value. Cells cultivated in KE medium pH 5.8 are always supplemented with 10mM lysine. Copy numbers of AdiY (+ *adiY*), CadC (+ *cadC*) or CsiR (+ *csiR*) were elevated by placing the corresponding genes under control of the arabinose (0.1%)-inducible promoter in plasmid pBAD24. All source data is summarized in Supplementary data 2.

gadC:eGFP-adiC:mCerulean-cadB:mCherry			
	pH 7.6	pH 5.8 + lysine	pH 4.4
t_0	GadC:eGFP<br>735 RF - 0.10<br><br>AdiC:mCerulean<br>35 RF - 0.04<br><br>CadB:mCherry<br>14 RF – 0.03		
t_{150}	GadC:eGFP<br>265 RF - 0.11<br><br>AdiC:mCerulean<br>42 RF - 0.05<br><br>CadB:mCherry<br>17 RF – 0.05	GadC:eGFP<br>1512RF - 0.04<br><br>AdiC:mCerulean<br>39 RF - 0.03<br><br>CadB:mCherry<br>386 RF – 0.37	
t_{300}	GadC:eGFP<br>836 RF - 0.11<br><br>AdiC:mCerulean<br>40 RF - 0.03<br><br>CadB:mCherry<br>15 RF – 0.04	GadC:eGFP<br>1734 RF - 0.08<br><br>AdiC:mCerulean<br>34 RF - 0.03<br><br>CadB:mCherry<br>246 RF – 0.26	GadC:eGFP<br>1401 RF - 0.05<br><br>AdiC:mCerulean<br>164 RF - 0.12<br><br>CadB:mCherry<br>547RF – 0.26

***E. coli* MG1655 wildtype**		
LB pH 4.4 t_{300}		
eGFP channel	mCerulean channel	mCherry channel
25 RF - 0.07	37 RF - 0.05	12 RF - 0.09

**<u>Δ*cadA*</u> *gadC:eGFP-adiC:mCerulean-cadB:mCherry* LB pH 4.4 t_{300}**		
GadC:eGFP<br>1848 RU - 0.06	AdiC:mCerulean<br>306 RU - 0.07	CadB:mCherry<br>1979 RU - 0.23

**<u>+ *csiR*</u> *gadC:mCerulean-cadB:mCherry* LB pH 4.4 t_{300}**	
GadC:mCerulean<br>1636 RF - 0.12	CadB:mCherry<br>495 RF - 0.29

**<u>+ *csiR*</u> *adiC:mCerulean-cadB:mCherry* LB pH 4.4 t_{300}**

<table>
<tr><td colspan="2">AdiC:mCerulean
92 RF - 0.10</td><td colspan="2">CadB:mCherry
509 RF - 0.31</td></tr>
<tr><td colspan="4"><u>+ cadC</u> gadC:mCerulean-cadB:mCherry KE pH 5.8 t_{300}</td></tr>
<tr><td colspan="2">GadC:mCerulean
856 RF - 0.05</td><td colspan="2">CadB:mCherry
1164 RF - 0.08</td></tr>
<tr><td colspan="4"><u>+ cadC</u> adiC:mCherry-cadB:eGFP LB pH 4.4 t_{300}</td></tr>
<tr><td colspan="2">AdiC:mCherry
30 RF - 0.08</td><td colspan="2">CadB:eGFP
1026 RF - 0.07</td></tr>
<tr><td colspan="4"><u>+ adiY</u> adiC:mCerulean-cadB:mCherry LB pH 4.4 t_{300}</td></tr>
<tr><td colspan="2">AdiC:mCerulean
333 RF - 0.06</td><td colspan="2">CadB: mCherry
396 RF - 0.29</td></tr>
<tr><td colspan="4"><u>gadC:mCherry</u>-adiC:mCerulean-cadB:eGFP</td></tr>
<tr><td>KE pH 7.6 t_{300}</td><td colspan="2">KE pH 5.8 t_{300}</td><td>LB pH 4.4 t_{300}</td></tr>
<tr><td>GadC:mCherry
767 RF - 0.12</td><td colspan="2">GadC:mCherry
1332 RF - 0.08</td><td>GadC:mCherry
1466 RF - 0.06</td></tr>
<tr><td colspan="4"><u>gadC:mCerulean</u>-adiC:mCherry-cadB:eGFP</td></tr>
<tr><td>KE pH 7.6 t_{300}</td><td colspan="2">KE pH 5.8 t_{300}</td><td>LB pH 4.4 t_{300}</td></tr>
<tr><td>GadC:mCerulean
680RF - 0.11</td><td colspan="2">GadC:mCerulean
1997 RF - 0.07</td><td>GadC:mCerulean
1532 RF - 0.09</td></tr>
</table>

Supplementary Table 2: Occurrence of the main components of the Gad, Adi and Cad system within selected species of the proteobacteria and firmicutes. Phylogenetic distribution is presented in Fig. 4 and sequences are summarized in Supplementary data 1. x, indicates present of a homolog of the protein GadA/B, GadC, CsiR, EvgS, GadE, GadW, GadY, YdeO, AdiA, AdiC, AdiY, CadA, CadB, CadC and LysP.

		Gad system									Adi system			Cad system			
		GadA/B	GadC	CsiR	EvgS	GadE	GadW	GadX	GadY	YdeO	AdiA	AdiC	AdiY	CadA	CadB	CadC	LysP
**Proteobacteria**	*Escherichia coli*	x	x	x	x	x	x	x	x	x	x	x	x	x	x	x	x
	Escherichia albertii	x	x	x		x	x	x	x		x	x	x	x	x	x	x
	Shigella boydii	x	x	x	x	x	x	x	x	x	x	x	x	x	x		x
	Shigella flexneri	x	x	x	x	x	x	x	x	x	x	x	x				
	Salmonella enterica			x							x	x	x	x	x	x	x
	Citrobacter freundii	x	x	x		x					x	x	x	x	x		x
	Hafnia alvei	x	x	x	x						x	x		x	x	x	x
	Serratia fonticola	x	x		x						x	x		x	x	x	x
	Xenorhabdus bovienii										x	x					x
	Stenotrophomonas maltophilia										x	x					
	Vibrio parahaemolyticus													x	x	x	
	Vibrio campbellii													x	x	x	
**Firmicutes**	*Clostridium perfringens*	x	x														x
	Lactococcus lactis	x	x														x
	Lactobacillus reuteri	x	x														x
	Listeria monocytogenes	x	x														x
	Enterococcus faecium	x	x														

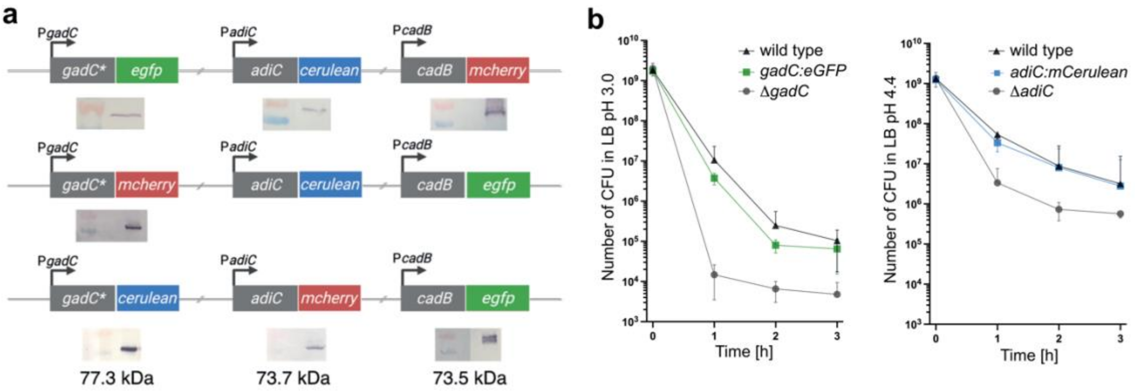

Supplementary Figure 1: Fluorescently-tagged antiporter hybrid proteins are functional in *E. coli*. (**a**) Schematic overview of the genomic organization of the different combinations of the three-color reporter strains: *gadC:eGFP-adiC:mCerulean-cadB:mCherry*, *gadC:mCherry-adiC:eGFP-cadB:mCerulean* and *gadC:mCerulean-adiC:mCherry-cadB:eGFP*. To verify the location of the different fluorescent hybrid proteins, the different three-color *E. coli* strains were pelleted at an OD_{600} = 1 at t_{300} in LB pH 4.4, fractionated and separated in a 12.5 % SDS-PAGE that was then transferred to nitrocellulose membrane. The different fluorescent hybrid proteins were labeled with either the primary polyclonal α-GFP antibody, α-mCerulean antibody or α-mCherry antibody and the α-rabbit alkaline phosphatase-conjugated antibody was used as secondary antibody. As ladder the PageRuler Prestained Protein Ladder (10 to 180 kDa) was used and only the membrane fraction is shown. The red band of the PageRuler Prestained Protein Ladder corresponds to 70 kDa. The molecular weight of each fluorescent hybrid protein is indicated below the Western blot. (**b**) Acid survival assay to test functionality of GadC:eGFP in LB medium pH 3.0 (left panel) and AdiC:mCerulean in LB medium pH 4.4 (right panel) via colony forming units during incubation for 3 h. The three-color strain *gadC:eGFP-adiC:mCerulean-cadB:mCherry* was cultivated and as control *E. coli* MG1655 cells and a deletion of either *gadC* or *adiC* were used. Functionality of the CadB:eGFP fusion was already assed via an liquid-based colorimetric assay using a pH indicator [1].

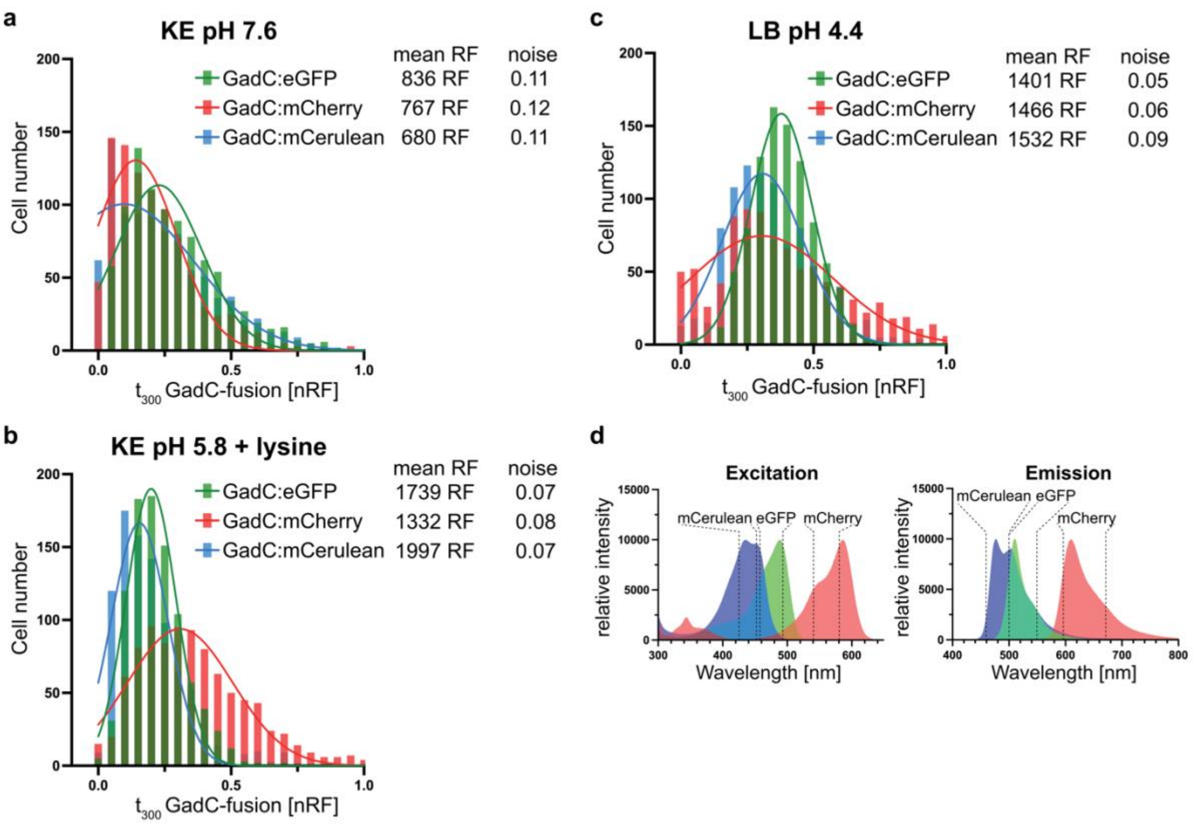

Supplementary Figure 2: Comparison of the production and distribution of the different fluorescent fusions with GadC at different acidic conditions. Histogram presentation of the normalized relative fluorescence intensity (nRF) quantified for 1,000 cells per fluorescent fusion, GadC:eGFP, GadC:mCherry or GadC:mCerulean, at t_{300} in (**a**) KE pH 7.6, (**b**) KE pH 5.8 supplemented with lysine and (**c**) LB pH 4.4. Calculated mean RF and noise values are summarized in Table S1. nRF, normalized RF values. (**d**) Comparison of the excitation (left panel) and emission (right panel) wavelength of the fluorophores mCerulean, eGFP and mCherry. Dashed lines indicate the ranges of the fluorescence filter cubes for the three fluorophores of the used Leica DMi8 inverted microscope. Spectra were obtained from the FPbase [2] and visualized using GraphPad Prism 9.1.0.

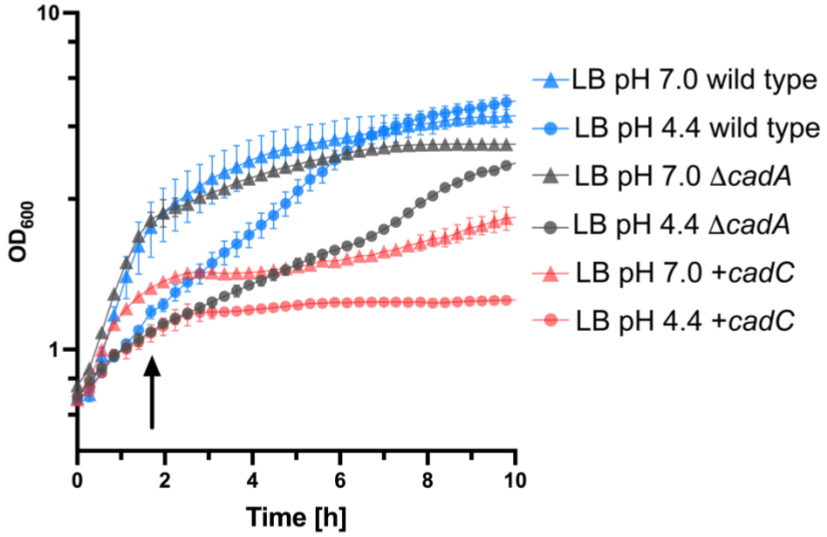

Supplementary Figure 3: High CadC copy number and *cadA* deletion slows down growth of *E. coli* at low pH. The *E. coli* strains, MG1655Δ*cadC* containing plasmid pBAD-*cadC,* and *E. coli* MG1655 wild type containing the empty plasmid pBAD24, were cultivated in KE medium pH 7.6 until exponential phase, then shifted to KE medium pH 5.8 + 10 mM lysine and finally shifted to LB medium at pH 7.0 or pH 4.4 (each supplemented with 0.1% (w/v) arabinose). Cultures were aerobically cultivated in 96-well plates at 37 °C in a Tecan Infinite F500 system (Tecan, Crailsheim, Germany) and growth (OD_{600}) was measured every 10 min at 37 °C. In strain MG1655Δ*cadC* + pBAD-*cadC*, the copy number of CadC is elevated to about 100 CadC molecules per cell, whereas the *E. coli* MG1655 pBAD24 strain produces only ≤4 CadC molecules per cell as reported previously [3]. The black arrow indicates the time point of the values displayed as a bar graph in Fig. 6b.

Supplementary References

1. Brameyer, S. *et al.* Molecular design of a signaling system influences noise in protein abundance under acid stress in different gammaproteobacteria. *J. Bacteriol.* 202, 95–15 (2020).

2. Lambert, T. J. FPbase: a community-editable fluorescent protein database. *Nat. Methods* 16, 277–278 (2019).

3. Ude, S. *et al.* Translation elongation factor EF-P alleviates ribosome stalling at polyproline stretches. *Science* 82–85 (2013).
