# Supplementary material for: Division of labor and collective functionality in Escherichia coli under acid stress
Source: Commun Biol. 2022 Apr 7;5:327. doi: 10.1038/s42003-022-03281-4 (PMC8989999; doi:10.1038/s42003-022-03281-4)
Supplement: Supplementary file 3 — Description of Additional Supplementary Files [file 42003_2022_3281_MOESM3_ESM.pdf]

Description of Additional Supplementary Files

File name: Supplementary Data 1: List of all GadC, AdiC and CadB homologs identified by a local alignment search based on the full-length sequence of E. coli GadC, AdiC and CadB.
Description: NCBI identifier numbers indicate the NCBI Pubmed identifier and are taken from the NCBI database for each GadC, AdiC or CadB homolog. The presence of the different regulatory components and the cognate decarboxylases in a certain species is indicated with the name of the component and the lack of the component is indicated with a “no”. GadC, AdiC or CadB homologs are separated in three different excel tabs.

File name: Supplementary Data 2
Description: List of all source data underlying the graphs and charts presented in the Figures 2, 3, 5 and 6.
